# Bladder malakoplakia mimicking a bladder tumor: A case report and brief review of the literature

**DOI:** 10.3892/mi.2025.292

**Published:** 2025-12-16

**Authors:** Rawa Bapir, Ismaeel Aghaways, Hadeel A. Yasseen, Ahmed H. Ahmed, Farman M. Faraj, Bryar Othman Muhammed, Twana Omer Saeed, Pavel W. Baqi, Marwan N. Hassan, Fahmi H. Kakamad

**Affiliations:** 1 Department of Scientific Affairs, Smart Health Tower, Sulaymaniyah 46001, Iraq; 2 Kscien Organization for Scientific Research (Middle East Office), Sulaymaniyah 46001, Iraq; 3 Department of Urology, Surgical Teaching Hospital, Sulaymaniyah 46001, Iraq; 4 College of Medicine, University of Sulaimani, Sulaymaniyah 46001, Iraq; 5 Smart Health Tower (Raparin Branch), Sulaymaniyah 46001, Iraq

**Keywords:** malakoplakia, urinary bladder, tumor mimicry, *Morganella morganii*

## Abstract

Malakoplakia is a rare granulomatous inflammatory disease that primarily affects the genitourinary tract and is often associated with coliform infections. In rare cases, it can mimic tumors; however, to date, to the best of our knowledge, no case of malakoplakia due to *Morganella morganii* mimicking as a tumor has been reported. The present study describes the case of a patient with bladder malakoplakia caused by *Morganella morganii*, mimicking a bladder tumor. A 78-year-old male patient presented with complaints of dysuria, urinary frequency, urgency and incontinence. Upon a urinalysis, a marked number of pus cells were observed, and an abdominal ultrasound revealed bladder wall thickening. Flexible cystoscopy revealed a tight urethra and multiple sessile masses in the bladder. A urine culture revealed infection with *Morganella morganii*, and the patient was treated with antibiotics. Following transurethral resection of the masses, histopathological analysis revealed von Hansemann cells and Michaelis-Gutmann bodies, confirming the diagnosis of malakoplakia. Immunohistochemistry was negative for AE1/AE3 and S100, but positive for CD68. In addition, 10 cases in the literature were reviewed; all patients in these cases were elderly or adults, apart from one case. Of note, 6 cases had *Escherichia coli* infection, and 1 case also had *Pseudomonas aeruginosa* infection. A total of 6 cases underwent mass resection, while 4 cases were managed conservatively; one of these experienced recurrence. On the whole, the present study demonstrates that bladder malakoplakia can also arise from infection with *Morganella morganii* and mimic a tumor; thus, it should be considered in the differential diagnosis of bladder inflammation or masses.

## Introduction

Malakoplakia is a rare inflammatory disease characterized by granuloma formation that commonly affects the genitourinary system (1). However, the disease can affect various anatomical locations; the bladder, renal parenchyma, prostate and ureter, are respectively, the most commonly affected areas (2). Other documented locations are the gastrointestinal tract, skin, epididymis, adrenal gland, bone and lungs, as well as multiple organs in rare conditions (3,4). Macroscopically, it often appears as soft, yellow-colored plaques or nodules. Although it usually appears as a single lesion, it can sometimes manifest as multiple lesions or may even be absent on a visual inspection (1). Malakoplakia can affect both sexes and is most common in individuals aged >40 years (2). The most common symptoms include hematuria, recurrent urinary tract infections and urinary flow obstruction (3). Although malakoplakia is an inflammatory condition, it can mimic a tumor, particularly when it appears as a mass-like lesion (5). The disease typically occurs in immunocompromised individuals, such as those with diabetes mellitus, kidney transplants, those with a prolonged use of systemic corticosteroids, or a history of *Escherichia coli* infection (2). However, to the best of our knowledge, no case of malakoplakia induced by *Morganella morganii* and mimicking a bladder tumor has been reported to date in the literature.

Therefore, the present study describes the case of a patient with bladder malakoplakia caused by *Morganella morganii* mimicking a bladder tumor. In line with the CaReL guidelines, a brief literature review was also included, and references were examined to ensure correct citations (6,7). A search was performed on Google Scholar using the key word ‘allintitle: Bladder Malakoplakia’ for publications dated between 2010 and 2025, yielding 37 results. From these, the 10 most recent cases were randomly selected for a brief review.

## Case report

### Patient information

A 78-year-old male patient presented to Smart Health Tower (Sulaymaniyah, Iraq) on December, 2024 with complaints of dysuria, associated with urinary frequency, urgency and incontinence, without hematuria. His past medical history included cerebrovascular accidents and hypertension. He had undergone a transurethral resection of the prostate in 2014 and had a femoral fracture resulting from a road traffic accident in 1990. His current medications included clopidogrel, rosuvastatin and amlodipine.

### Clinical findings

Upon a general examination, the patient appeared alert and oriented with no acute distress. He relied on a wheelchair for mobility assistance. His vital signs were within normal limits. The abdominal examination revealed a soft, non-tender abdomen with no palpable masses or suprapubic fullness. External genitalia appeared normal with no signs of erythema, discharge, or lesions.

### Diagnostic approach

**Figure 1:**
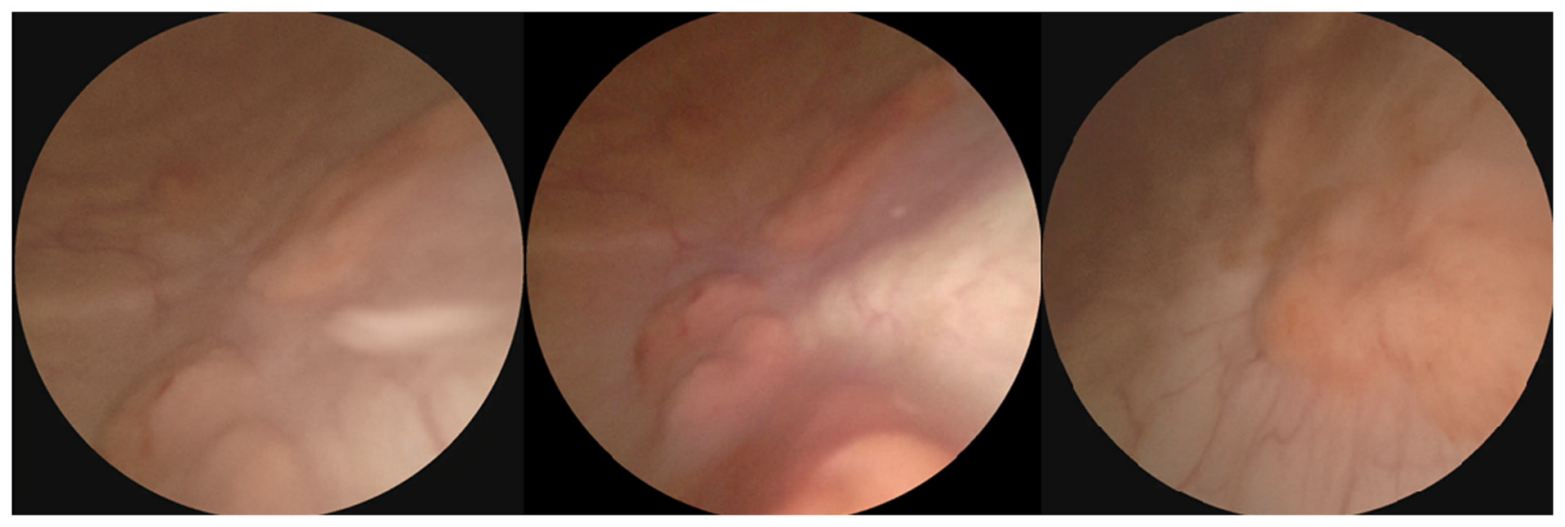
Cystoscopy images (all three images) illustrating multiple, diffuse, irregular and sessile masses throughout the bladder.

A urinalysis revealed a marked number of pus cells per high-power field. An abdominal ultrasound demonstrated a thickened bladder wall, indicative of cystitis. A urine culture revealed the growth of *Morganella morganii*. At 20 days thereafter, the patient returned with similar symptoms despite antibiotic use [cefditoren pivoxil, 200 mg (1x2) for 3 weeks]. A repeated urinalysis revealed similar findings. A subsequent flexible cystoscopy revealed a tight urethra and multiple diffuse sessile masses throughout the bladder (Fig. 1).

### Therapeutic intervention

**Figure 2:**
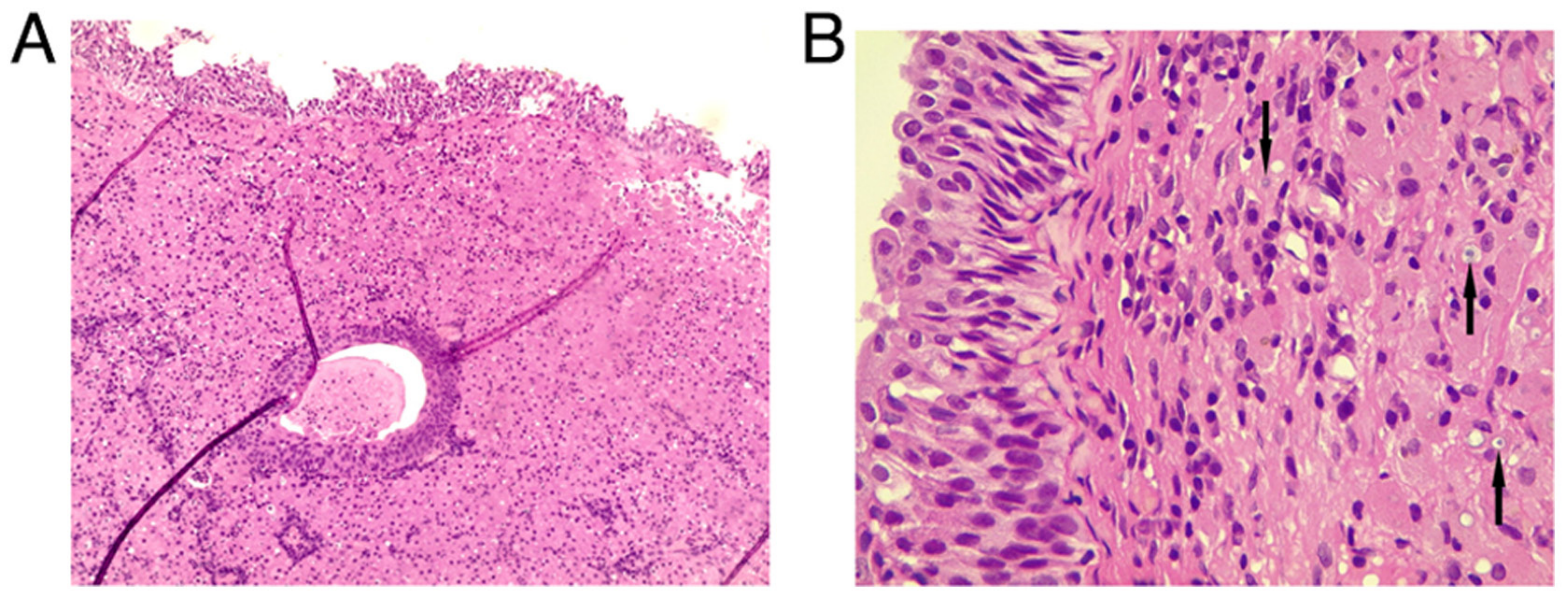
(A) Subepithelial mucosal infiltrate composed of sheets of polygonal cells with granular eosinophilic cytoplasm (magnification, x40). (B) Some polygonal cells display basophilic intracytoplasmic inclusions (arrows); hematoxylin and eosin staining (magnification, x100).

**Figure 3:**
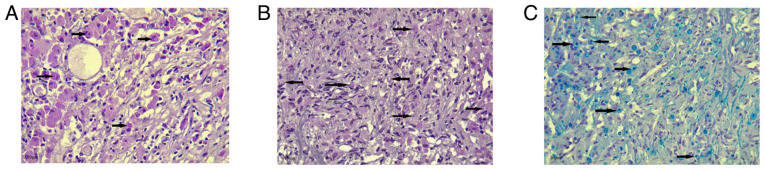
(A) Periodic acid-Schiff staining highlighting the intracytoplasmic inclusions (arrows) (magnification, x100). (B) Periodic acid-Schiff with diastase staining demonstrating the presence of Michaelis-Gutmann bodies (arrows) (magnification, x100). (C) Prussian blue staining illustrating the Michaelis-Gutmann bodies (arrows) (magnification, x100).

**Figure 4:**
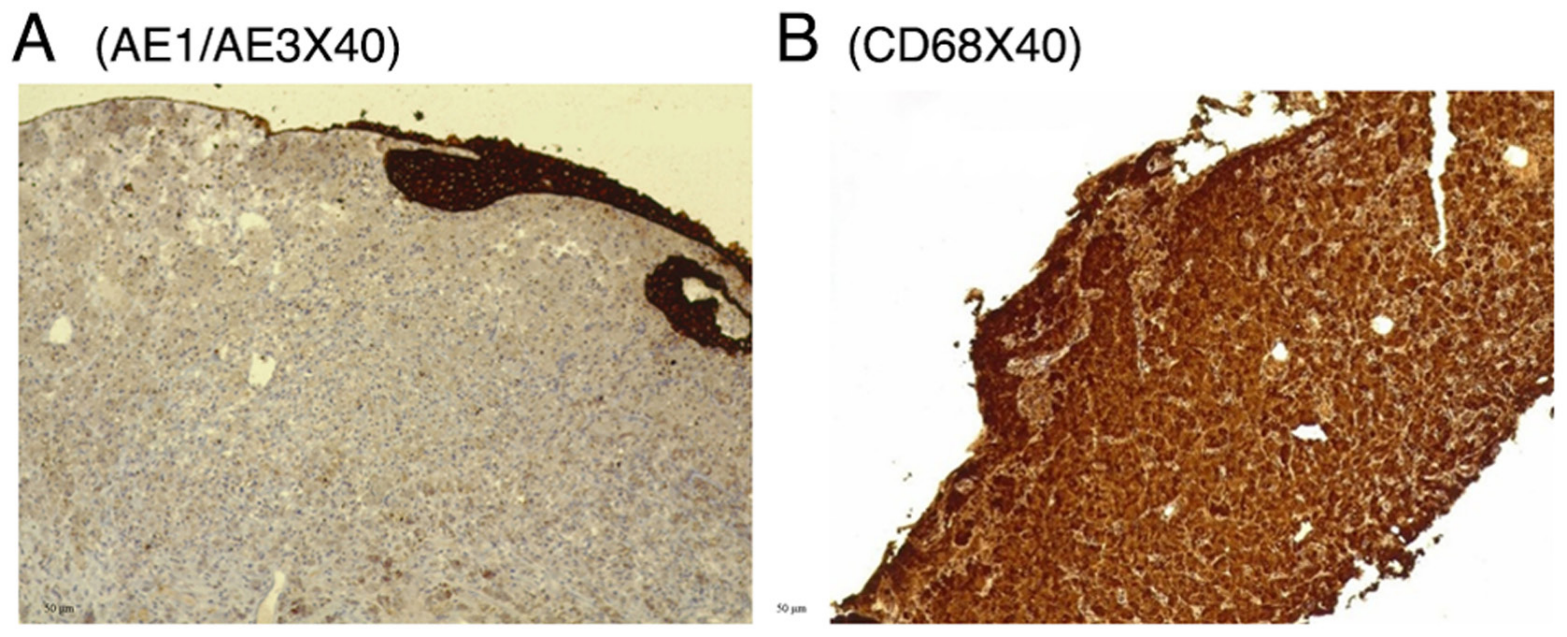
(A) Mucosal polygonal cells are negative for AE1/AE3, effectively excluding an epithelial origin (magnification, x40). (B) Strong, diffuse cytoplasmic positivity for CD68 supports a histiocytic lineage (magnification, x40).

A transurethral resection of the masses was performed and sent for histopathological analysis. Grossly, several rubbery, gray-tan tissue fragments measuring 2.5 cm in total were noted. A histopathological examination, was then performed on 5-µm-thick paraffin-embedded sections. The sections were fixed in 10% neutral buffered formalin at room temperature for 24 h and then stained with hematoxylin and eosin (Bio Optica Co.) for 1-2 min at room temperature. The sections were then examined under a light microscope (Leica Microsystems GmbH). Histological analysis revealed a reactive, flattened urothelial lining with mild hyperplasia, featuring focal Brunn nests and cystitis glandularis. There was a marked mucosal infiltrate composed of sheets of round to polygonal histiocyte cells with abundant eosinophilic granular cytoplasm, resembling von Hansemann cells (Fig. 2). These cells had round nuclei with variable nucleoli and displayed rounded, concentric basophilic intracytoplasmic inclusions, Michaelis-Gutmann-like bodies. The tissue also contained a mixture of acute and chronic inflammation. The muscularis propria was present and unaffected by the histiocytic infiltrate. Prussian blue, Periodic acid-Schiff (PAS) and PAS with diastase (D-PAS) staining was performed on 1.5-µm-thick paraffin-embedded sections. The sections were fixed in 10% neutral buffered formalin at room temperature for 24 h. Prussian blue, PAS and D-PAS staining (MilliporeSigma) were then applied at room temperature for a duration of 20 min. The sections were then examined under a light microscope (Leica Microsystems GmbH). Prussian blue, PAS and D-PAS staining all yielded positive results, highlighting the cytoplasmic inclusion (Fig. 3). Immunohistochemistry (IHC) was then performed for mucosal infiltrating cells. Serial 5-µm-thick sections cut from formalin-fixed, paraffin-embedded bladder mass tissue were mounted on coated slides, deparaffinized in xylene and rehydrated through graded ethanol to distilled water. Antigen retrieval was performed by heat-mediated epitope retrieval in 10 mM citrate buffer (Ph 6.0) at 95-100˚C for 20 min, followed by cooling for 20 min at room temperature. Endogenous peroxidase activity was quenched with 3% hydrogen peroxide in PBS for 10 min at room temperature. Sections were permeabilized with 0.1% Triton X-100 in PBS for 10 min at room temperature and rinsed with PBS (3x5 min). Non-specific binding was blocked with 5% normal goat serum (Thermo Fisher Scientific, Inc.) in PBS for 20 min at room temperature. Primary antibodies [AE1/AE3 (1:100, clone AE1/AE3, cat. no. M3515), S100 (1:200, polyclonal rabbit, cat. no. Z0311) and CD68 (1:100, clone KP1, cat. no. M0814) (all from Dako; Agilent Technologies, Inc.)] were applied and incubated with the sections for 30-60 min at room temperature (alternatively overnight at 4˚C if required for optimization). Following washes with distilled water, the sections were incubated with HRP-conjugated goat anti-mouse IgG (1:200, cat. no. P0447) for AE1/AE3 and CD68, and HRP-conjugated goat anti-rabbit IgG (1:200, cat. no. P0448) for S100 (both from Dako; Agilent Technologies, Inc.), for 30 min at room temperature. HRP activity was visualized using DAB chromogen according to manufacturer's instructions (monitoring 3-10 min), followed by a water rinse. The sections were counterstained with Gill's hematoxylin II (Thermo Fisher Scientific, Inc.) for ~30-60 sec, rinsed, blued in running tap water, dehydrated, cleared and coverslipped. Images were captured on a Leica light microscope (Leica Microsystems GmbH); scale bars (50 µm) were added following objective calibration. IHC revealed negativity for AE1/AE3 and S100 (data not shown), and strong positivity for CD68 (Fig. 4). Negativity for AE1/AE3 argued against an epithelial cell origin, while negativity for S100 argued against a granular cell tumor. Positivity for CD68 supported the presence of histiocytic-type cells. The patient was administered oral sulfamethoxazole-trimethoprim (800/160 mg) twice daily for 5 days. Following the urine culture results, treatment was switched to intravenous meropenem (1 g) twice daily for 4 days. No side-effects from the treatment were observed.

### Follow-up and outcome

Following antibiotic therapy, the symptoms of the patient partially improved at 1-month follow-up. The patient was subsequently lost to follow-up.

## Discussion

When involving the genitourinary system, malakoplakia is more commonly observed in women (8). However, malakoplakia outside the urinary tract is more common among males and is uncommon in children (3). The precise cause of malakoplakia is not yet fully understood. It is hypothesized that malakoplakia results from impaired phagocytosis, linked to reduced levels of intracellular cyclic guanosine monophosphate. A low cyclic guanosine monophosphate to cyclic adenosine monophosphate ratio disrupts the redox balance of the cell, compromising its ability to kill bacteria effectively. This leads to a granulomatous response, where bacterial remnants accumulate. These partially digested bacteria calcify and cluster inside macrophages, forming the characteristic Michaelis-Gutmann bodies (5). While a direct cause-and-effect connection between coliform bacteria and malakoplakia has not yet been firmly established, it has been reported that ~90% of patients with malakoplakia also have coliform infections (8).

*Morganella morganii*, a bacterium from the *Enterobacteriaceae* family, is a rod-shaped, Gram-negative, facultatively anaerobic bacillus that includes two subspecies: *Morganii and sibonii*. Formerly known as *Proteus morganii*, it is typically part of the normal human gut microbiota. However, in certain cases, particularly in hospital settings, following surgery, or in individuals with weakened immune systems and young children, it can lead to severe and potentially life-threatening systemic infections, and naturally exhibits resistance to several antibiotic classes (9). Herein, following a detailed literature review, no cases of malakoplakia caused by *Morganella morganii* were identified. In addition, Polisini *et al* (2) reviewed the literature on bladder malakoplakia and, across 35 articles reporting 36 cases, did not identify any case of bladder malakoplakia associated with *Morganella morganii*. Hence, the case presented herein may be the first reported case of its kind.

Polisini *et al* (2) reported that approximately half of the cases had recurrent urinary tract infections, particularly caused by *Escherichia coli*, and that ~20% had immune system disorders. Antibiotic therapy was administered in ~70% of cases, and surgery was performed in 67% of cases. Among the surgical procedures, approximately three-quarters involved transurethral resection of the bladder. The recurrence rate was lower in patients managed with both antibiotics and surgery than in those treated with antibiotics alone (2). Malakoplakia can also occur in unusual sites, such as the brain and skeleton, which may indicate atypical *Escherichia coli* strains or immune system abnormalities (3). A total of 10 cases of bladder malakoplakia were reviewed herein (1,3,4,8,10-15). All cases involved older individuals or adults, apart from 1 patient who was <2 years of ag. Michaelis-Gutmann bodies were identified in all histopathological examinations, with 2 cases also reporting von Hansemann cells. Among the cases that performed urine cultures, only 2 cases were negative, while the others were infected with *Escherichia coli*; 1 case also had a positive blood culture for *Pseudomonas aeruginosa*. Surgical intervention was performed in 6 cases, while 4 cases were managed conservatively, one of which experienced recurrent bladder malakoplakia after 6 months (Table I).

There may be instances in which malakoplakia occurs without clinical or laboratory evidence of infection, but rather in association with underlying neoplastic conditions. Darvishian *et al* (16) reported the case of a 74-year-old female patient with papillary urothelial carcinoma of the urinary bladder, with malakoplakia identified incidentally in a bladder biopsy. Urinalysis revealed only a marked presence of numerous red blood cells, with no evidence of pyuria or bacteriuria. The urine culture did not reveal bacterial growth. A tumor on the lateral wall of the urinary bladder was noted in cystoscopy following initial examinations, and a biopsy was subsequently done. Histological analysis and immunohistochemistry were performed, exhibiting CD68 positivity. She underwent conservative treatment without additional invasive intervention (16).

There is currently no universally accepted standard for the diagnosis and treatment of malakoplakia (5). Since the majority of studies on bladder malakoplakia are case reports, with a notable absence of comprehensive reviews (2), diagnosing malakoplakia clinically can be challenging due to its non-specific features. However, its appearance may mimic carcinoma, which often leads to invasive or minimally invasive treatment, as in the present case report, since malakoplakia generally requires conservative treatment (17).

Its histological morphology changes over three stages. In the first stage, a few cells with lymphocyte and plasma cell infiltration represent glucolipid aggregation in Michaelis-Gutmann bodies. The second stage is marked by increased histiocytes and von Hansemann cells, along with the formation of Michaelis-Gutmann bodies, which is the most distinctive feature. In the third stage, histiocytes and von Hansemann cells decrease, and fibrosis and collagen hyperplasia occur. Stains such as PAS, Prussian blue, Alcian Blue and von Kossa effectively highlight Michaelis-Gutmann bodies (18). Increased levels of immunoreactive α1-antitrypsin are observed in macrophages in malakoplakia, potentially aiding in the differential diagnosis. Malakoplakia also presents with non-specific imaging findings, and the affected areas may develop tumor-like nodules (5). Still, imaging techniques, such as computed tomography, ultrasonography and intravenous pyelogram can help detect associated hydroureteronephrosis and identify upper urinary tract filling defects, which are suggestive of its presence (2). Immunohistochemistry also aids in diagnosing malakoplakia by exhibiting negativity for AE1/AE3 and S100, and positivity for CD68(3). Immunohistochemistry staining in the present case report revealed negativity for AE1/AE3 and S100, and positivity for CD68, similar to has been previously reported (3).

The treatment of malakoplakia depends on the severity of the condition and the underlying health conditions of the patient. In the majority of cases, patients with bilateral or multifocal involvement recover following antibiotic therapy. A cholinergic agonist such as bethanechol chloride may be used alongside antibiotics to help correct lysosomal dysfunction (17). There are no established guidelines regarding selection or duration of antibiotic therapy to prevent malakoplakia recurrence. However, the use of antibiotics that are effective against Gram-negative bacteria and those that accumulate within macrophages has been recommended, as they may help compensate for impaired phagocytic function. These antibiotics include rifampicin, ciprofloxacin and trimethoprim. Due to the potential adverse effects associated with quinolones, trimethoprim is often the preferred option (7). Vitamin C supplementation is recommended to help reduce the inflammatory response. In patients with malakoplakia-related complications, the regular monitoring of renal function and ongoing cystoscopic surveillance are essential (10). Surgical removal has also been recommended as an alternative treatment option for large lesions, especially when complete elimination through medical therapy alone may not be possible (8). Unifocal disease may require surgical excision, and transurethral resection may be needed for large lesions blocking the ureter. Based on the risk-benefit ratio, the termination of immunosuppressive drugs is often necessary (3). The patient in the present case report was managed using a combination of transurethral resection of the mass-like lesions and antibiotic therapy. The patient demonstrated a favorable initial response with a partial improvement of symptoms; however, the case was lost to follow-up, preventing the further assessment of disease progression. Consequently, the major limitation of the present case report is the short follow-up period, which restricts the ability to accurately evaluate the long-term outcome of the patient. In addition, there is a lack of a representative image for S100 reactivity. The present study presents only a single-center case; thus, further research is warranted to elucidate the potential role and underlying mechanisms of *Morganella morganii* infection in the pathogenesis of malakoplakia and to provide deeper insights into this association.

In conclusion, bladder malakoplakia can also develop due to infection with *Morganella morganii* and mimic a tumor; thus, it should be considered in the differential diagnosis of bladder inflammation or masses.

## Figures and Tables

**Table I:** Review of 10 cases of malakoplakia in the urinary bladder identified in the literature.

First author, year of publication	Sex	Age (years)	Chief complaint/symptoms	Imaging tests	Histopathological and immunohistochemical examination	Culture tests	Other examinations	Treatment	Follow-up	(Refs.)
Ye, 2025	F	N/A	Recurrent urinary frequency for >1 year	CT: Intravesical mass and upper ureteral calculi Cystoscopy: A mass at bladder neck and urethral opening	HPE: Michaelis-Gutmann bodies	N/A	N/A	Transurethral bladder tumor resection	Not mentioned.	(11)
Tinguria, 2024	F	86	Nausea, diarrhea, foul-smelling urine, dysuria, hematuria, and low blood pressure	CT: Atrophy of the left kidney, & diffuse bladder wall thickening.	HPE: Foamy epithelioid histiocytes, PAS-positive eosinophilic, calcified Michaelis-Gutmann bodies. IHC: +ve vimentin and CD-68, -ve AE1/AE3.	Urine: +ve *E. coli*, and *P. aeruginosa*, blood: +ve *P. aeruginosa.*	CR: 252 µmol/l, troponin: 2,695 ng/l, WBC count: 15.4x10^9^/l, cystoscopy: Plaque-like whitened lesions	Piperacillin/tazobactam,	Recurrent bladder malakoplakia after 6 months.	(3)
Jdiaa, 2023	F	70	Fatigue, weakness, decreased oral intake	CT: Bilateral ureteral fullness suspicious for urothelial malignancy, bilateral hydronephrosis	HPE and IHC: Sheets of epithelioid histiocytes (CD68 positive), numerous neutrophils, numerous CD38-positive plasma cells and occasional Michaelis-Gutmann bodies	Blood and urine: +ve *E. coli*	CR: 5.0 mg/dl Hemoglobin: 6.9 g/dl WBC: 13.9x10^9^/l	Transurethral bladder tumor resection, ceftriaxone	Recovery with return of kidney function to baseline	(12)
Gao, 2021	M	48	Frequent and urgent urination, right lumbago swelling and pain, as well as lower abdominal discomfort	CT: A mass on the right lateral bladder wall invading the right ureteral orifice	HPE: large numbers of eosinophils and foam cells containing Michaelis-Gutmann bodies	Urine: +ve *E. coli*	ESR: 98 mm/h CR: 123 µmol/l	Transurethral bladder mass resection, tazobactam	No recurrence after 4 years	(13)
Pham, 2022	F	81	Frequent and urgent urination, Urge incontinence	CT: Severe bilateral hydrou-reteronephrosis and bladder wall thickening	HPE: Michaelis-Gutmann bodies	N/A	CR: 221 µmol/l	Cephalexin, methenamine hippurate, supplemental vitamin C, ntravesical gentamicin wash	Not mentioned	(14)
Parkin, 2020	F	82	History of T1D, rigors, lethargy, back pain, irritative voiding and urgency	CT: Nilateral hydroureterone-phrosis and marked thickening of the bladder base	HPE: Michaelis-Gutmann bodies & von Hansemann cells. No evidence of dysplasia or malignancy was seen.	Urine: recurrent pan-sensitive *E. coli.*	WBC count: 5.4×10^9/l, hemoglobin: 96 g/l & CR: 234 µmol/l.	Surgical removal of the bladder mass, amoxicillin-clavulanic acid	No recurrence at 2 months.	(10)
Rabani, 2019	F	1.7	voiding dysfunction, pyuria & repeated UTI.	CT: right lateral bladder wall mass.	HPE: Michaelis-Gutmann bodies.	Urine: +ve *E. coli.*	Normal blood profile, renal, and liver function tests. Cystoscopy: bladder mass.	Trimethoprim-sulfamethoxazole	No recurrence after 9 years.	(8)
Sirithanaphol, 2018	F	66	Gross hematuria, dysuria	Cystoscopy: white-yellowish plaque in bladder	HPE: sheets of large macrophages with granular eosinophilic cytoplasm, mixed inflammatory infiltrate, and Michaelis-Gutmann bodies	Urine: -ve	Urinalysis: Pyuria and hematuria	Ciprofloxacin, prednisolone, methotrexate, trimethoprim-sulfamethoxazole	Recovery of symptoms	(15)
Lee, 2015	F	74	Chills, unremarkable physical examination.	CT: urinary bladder postero-lateral wall mass.	HPE: papillary urothelial carcinoma, & macrophages with Michaelis-Gutmann bodies. IHC: +ve CD163 & CD68 & -ve Cam 5.2.	Urine: -ve.	WBC count: 12.9x10^3/µl, CRP: 77.4 mg/l, PCT: 64.28 µg/l CR: 101 µmol/l, Urinalysis: hemopyuria.	IV ceftriaxone for possible sepsis, transu-rethral resection	No recurrence at 12-month follow-up.	(1)
Ristić-Petrović, 2013	F	53	General weakness, low-grade fever and urinary hesitancy.	Not mentioned	HPE: von Hansemann cells, Michaelis-Gutmann bodies, infiltrated by dense collections of lymphocytes. IHC: -ve cytokeratin, and Ki-67, +ve CD68	Persistent *E. coli* infections for 2 years.	Urine analysis: Albuminuria, macrohematuria, pyuria and bacteriuria. Cystoscopy: Thickened mucosa of the bladder similar to a neoplastic mass.	Surgical removal of yellowish polypoidal lesions of the bladder	Not mentioned	(4)

N/A, not available; F, female; M, male; WBC, white blood cell; CRP, C-reactive protein; CR, creatinine; ESR, erythrocyte sedimentation rate; PCT, procalcitonin; +ve, positive; -ve, negative; *E. coli*, *Escherichia coli; P. aeruginosa*, *Pseudomonas aeruginosa*; CT, computed tomography scan; IHC, immunohistochemistry; HPE, histopathological examination; IV, intravenous; UTI, urinary tract infection; T1D, type one diabetes.

## Data Availability

The data generated in the present study may be requested from the corresponding author.
